# β-Cyclodextrin/Triclosan Complex-Grafted Methacrylated Glycol Chitosan Hydorgel by Photocrosslinking via Visible Light Irradiation for a Tissue Bio-Adhesive

**DOI:** 10.3390/ijms22020700

**Published:** 2021-01-12

**Authors:** Young Jae Moon, Sun-Jung Yoon, Jeung-Hyun Koo, Yihyun Yoon, Hye Jun Byun, Hyeon Soo Kim, Gilson Khang, Heung Jae Chun, Dae Hyeok Yang

**Affiliations:** 1 Department of Biochemistry & Molecular Biology & Orthopaedic Surgery, Research Institute for Endocrine Sciences, Jeonbuk National University Hospital, Jeonbuk National University Medical School, Jeonju 54896, Korea; yjmoonos@jbnu.ac.kr (Y.J.M.); jhyuni81@jbnu.ac.kr (J.-H.K.); 2 Department of Orthopedic Surgery, Research Institute of Clinical Medicine of Jeonbuk National University, Biomedical Research Institute of Jeonbuk National University Hospital, Jeonbuk National University Medical School, Jeonju 54896, Korea; sunjungyoon@jbnu.ac.kr; 3 Institute of Cell and Tissue Engineering, College of Medicine, The Catholic University of Korea, Seoul 06591, Korea; 21903676@cmcnu.or.kr (Y.Y.); 21904860@cmcnu.or.kr (H.J.B.); 21905010@cmcnu.or.kr (H.S.K.); chunhj@catholic.ac.kr (H.J.C.); 4 Department of BIN Convergence Technology & Polymer Nano Science & Technology and Polymer BIN Research Center, Jeonbuk National University, Jeonju 54896, Korea; gskhang@jbnu.ac.kr; 5 Department of Biomedical & Health Sciences, College of Medicine, The Catholic University of Korea, Seoul 06591, Korea

**Keywords:** methacrylate glycol chitosan, beta-cyclodextrin, triclosan, antibacterial, tissue bio-adhesive

## Abstract

Accelerating wound healing with minimized bacterial infection has become a topic of interest in the development of the new generation of tissue bio-adhesives. In this study, we fabricated a hydrogel system (MGC-g-CD-ic-TCS) consisting of triclosan (TCS)-complexed beta-cyclodextrin (β-CD)-conjugated methacrylated glycol chitosan (MGC) as an antibacterial tissue adhesive. Proton nuclear magnetic resonance (^1^H NMR) and differential scanning calorimetry (DSC) results showed the inclusion complex formation between MGC-g-CD and TCS. The increase of storage modulus (G’) of MGC-g-CD-ic-TCS after visible light irradiation for 200 s indicated its hydrogelation. The swollen hydrogel in aqueous solution resulted in two release behaviors of an initial burst and sustained release. Importantly, in vitro and in vivo results indicated that MGC-g-CD-ic-TCS inhibited bacterial infection and improved wound healing, suggesting its high potential application as an antibacterial tissue bio-adhesive.

## 1. Introduction

Self-healing tissue adhesives have been regarded as attractive sealants for wound closure in surgery and trauma handling because they perform several important functions including promoting wound healing acceleration by preferably binding to tissues, as well as sealing leaks and stopping tissue adhesion [1]. However, cyanoacrylate-based adhesives are very toxic due to the formaldehyde that result from their degradation, and fibrin glues have weak tensile and adhesive strength, and problems with viral contamination [2,3]. Therefore, new types of tissue adhesives that overcome the drawbacks of the existing adhesives should be developed.

Hydrogels are promising candidates as self-healing tissue adhesives, since damaged wounds can be regenerated by their hydrophilic and crosslinked structures, such as soft tissues, and high permeability to oxygen, nutrients, and water-soluble metabolites [1]. In addition, hydrogels that can load drugs and proteins allow for the improvement in tissue regeneration [1,4]. In particular, antibacterial agent-containing hydrogels are currently a main focus in biomedical research and applications such as a surgical-site infection, because infection is a critical issue for successful wound healing [5,6]. Among the available antibacterial agents, triclosan (TCS) is a commonly used drug because of its broad spectrum of antibacterial activity, and TCS is widely used from industry to medical fields [7]. In medical fields, a TCS-coated suture is being used clinically.

Among the basic materials for hydrogels, water-soluble chitosan derivatives are coming into the spotlight as self-healing tissue adhesives [8,9]. These chitosan adhesives are potentiated by hydrogen bonding and electrostatic interaction between the polymer and collagen fibers [10]. The chitosan derivatives can be easily designed in the hydrogel form under an aqueous condition because amine groups in the polymer backbones have functional groups by various modification techniques, followed by hydrogel formation by various stimuli such as light, chemical, and temperature stimuli [11,12,13,14,15,16]. Ultraviolet/visible light-curing systems are efficient tools for preparing hydrogel tissue adhesives because they can be controlled by manipulating the presence and dose of light, and independence of spatial or temporal control of different biological processes in a wavelength-specific manner [17,18,19]. Notably, a visible light-curing system is safer than a system using ultraviolet light, because ultraviolet light can lead to melanoma and suppress the function of the immune system [20].

**Figure 1:**
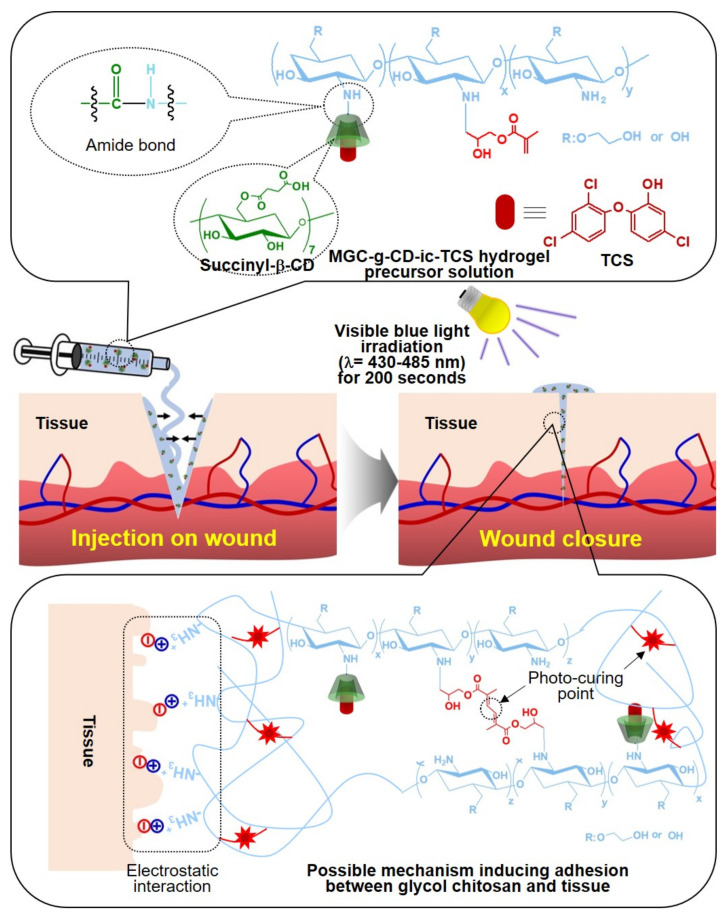
Schematic illustration on the application of β-cyclodextrin/triclosan complex-grafted methacrylated glycol chitosan (MGC-g-CD-ic-TCS) as a tissue bio-adhesive and possible mechanism of adhesion between MGC-g-CD-ic-TCS and soft tissue.

In this work, we prepared a self-healing hydrogel tissue bio-adhesive (MGC-g-CD-ic-TCS) with antibacterial activity based on visible light-cured methacrylated glycol chitosan (MGC) and succinyl-beta-cyclodextrin/triclosan (β-CD-ic-TCS) for wound closure. The efficacy was evaluated using a rat model of skin incision (Figure 1). A methacrylic group and succinyl-β-CD (β-CD-COOH) were conjugated to water-soluble GC for a visible light-curing system (MGC-g-CD). The water solubility of TCS was improved by host-guest inclusion complex with MGC-g-CD, which was characterized by proton nuclear magnetic resonance (^1^H NMR) and differential scanning calorimetry (DSC) analyses. The MGC bio-adhesive was prepared using a blue visible light irradiation with riboflavin as a photoinitiator, which were characterized using rheology, in vitro drug release, in vitro cytotoxicity, and antibacterial activity assays. The tissue adhesion properties of MGC-g-CD-ic-TCS were compared with those of commercially used adhesives such as fibrin glue and cyanoacrylate. In addition, the self-healing capacity of MGC-g-CD-ic-TCS was compared with that of untreated incision, suture, cyanoacrylate, and MGC hydrogel.

## 2. Results and Discussion

### 2.1. ^1^H NMR Spectra of MGC-g-CD and MGC-g-CD-ic-TCS

**Figure 2:**
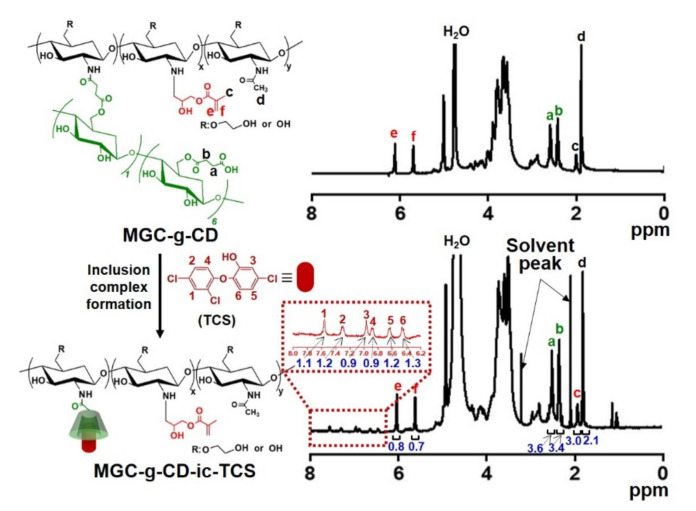
^1^H NMR spectra of MGC-g-CD and MGC-g-CD-ic-TCS analyzed using D_2_O. a and b—CH2CH2- peak of succinyl β-CD; c, e, and f-methyl and vinyl groups of glycidyl methacrylate; d-methyl group of glycol chitosan. The numbers from 1 to 6 in the dotted line indicated triclosan.

Conjugation of succinyl-β-CD to the MGC backbone and the inclusion complex between MGC-g-CD and TCS were confirmed by ^1^H NMR analysis in D_2_O (Figure 2). We previously reported the successful preparation of MGC formed by reaction between GC and glycidyl methacrylate through ^1^H NMR analysis using D_2_O [11,12,13,14]. The ^1^H NMR spectrum of MGC exhibited newly formed peaks at 2.02 ppm, 5.69 ppm, and 6.11 ppm assigned for the methyl and vinyl groups of methacrylation. Consistent with our previous reports [11,12,13,14], the ^1^H NMR spectrum of MGC-g-CD also showed peaks related to methacrylation at the same locations, indicating formation of MGC. The degree of substitution (DS) of the methyl group was calculated in consideration of the integration ratio between the glucopyranosyl ring peak area of GC at 3.23–4.21 ppm and the -CH_3_ peak area of the methacrylic group at 1.89 ppm, resulting in a methacrylate of 70%. MGC-g-CD produced new peaks at 2.42 ppm and 2.59 ppm, and 5.00 ppm, which were assigned for -CH_2_CH_2_- of the succinyl group and H-1 of the glucopyranosyl group, respectively [21,22]. In addition, the DS of β-CD calculated from the integration ratio between the glucopyranosyl ring peak at 3.23-4.21 ppm and the -CH_2_- peak area of succinyl group at 2.34 ppm was 24%. Inclusion complex formation between MGC-g-CD and TCS made the appearance of new peaks possible, because ^1^H NMR analysis of poorly water-soluble TCS is impossible in D_2_O. The peaks of complexed TCS were observed at 6.45 ppm, 6.64 ppm, 6.88 ppm, 6.96 ppm, 7.30 ppm, and 7.56 ppm. In comparison with the -CH_2_CH_2_- peak of the succinyl group, the inclusion complex percentage of TCS was almost 100%.

### 2.2. DSC Curve and Storage/Loss Moduli of MGC-g-CD-ic-TCS

**Figure 3:**
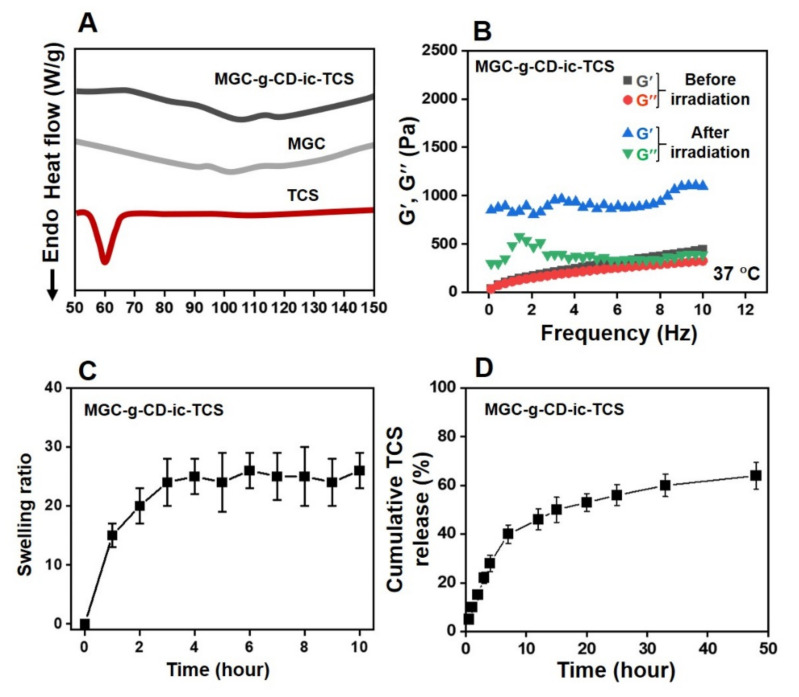
(**A**) DSC curves of triclosan (TCS), methacrylated glycol chitosan (MGC), and MGC-g-CD-ic-TCS monitored from 50 °C to 150 °C, (**B**) storage/loss moduli of MGC-g-CD-ic-TCS hydrogel precursor solution measured from 0 Hz to 10 Hz before and after visible light irradiation for 200 s, (**C**) swelling ratio of MGC-g-CD-ic-TCS measured at determined time intervals (0, 1, 2, 3, 4, 5, 6, 7, 8, 9, and 10 h), (**D**) Cumulative TCS release percentage calculated at determined time intervals (0.5, 1, 2, 3, 4, 7, 12, 15, 20, 25, 33, and 48 h). Swelling ratio and release behavior tests were performed in triplicate (*n* = 3).

To further investigate the inclusion complex formation between MGC-g-CD and TCS, DSC was employed and compared with the results of MGC-g-CD and TCS (Figure 3A). TCS possesses a strong endothermic peak around 59 °C [23]. In a previous study, if an inclusion complex between cyclodextrins and TCS was successfully formed, the endothermic peak of TCS disappeared [23]. As shown in Figure 3A, MGC-g-CD-ic-TCS did not show the endothermic peak around 59 °C. Based on the previous study, we suggest that TCS is included into the ring molecules of MGC-g-CD-ic-TCS without uncomplexed TCS.

The storage modulus of MGC-g-CD-ic-TCS hydrogel precursor solution before and after visible light irradiation for 120 s was monitored from 0 Hz to 10 Hz at 37 °C (Figure 3B). Visible light irradiation enhanced the storage modulus of the precursor solution. Before irradiation, the storage/loss moduli at 0 Hz and 10 Hz were 0/0 Pa and 440/326 Pa, respectively, but the two frequencies of the sample after irradiation were 850/300 Pa and 1098/392 Pa, respectively. This improved storage modulus may be from a three-dimensional (3-D) network formed from photocuring among the methacrylic groups of MGC-g-CD. This 3-D structure has a significant influence on the release behavior of hydrophobic triclosan [24]. Hydrogels exhibit controlled and sustained release of hydrophobic drugs [24]; this release behavior is closely related to the swelling property of hydrogels. Because of the open spaces in the polymer chains of hydrogels, the matrices allow for the absorption of solute and drugs and the diffusion of the drugs [25]. In addition, β-CD conjugated to polymer backbones in a hydrogel matrix makes it easier to release hydrophobic drugs [26]. Here, we conducted the inclusion complex formation between MGC-g-CD and TCS, and the swelling ratio and release test were evaluated (Figure 3C,D). The results were in accordance with the correlation between the swelling ratio and release behavior of hydrogels. MGC-g-CD-ic-TCS hydrogel was swollen by 3 h due to the migration of water molecules. Since then, the hydrogel remained maintained an equilibrium state for 24 h (Figure 3C). Figure 3D shows the release behavior of TCS from MGC-g-CD-ic-TCS over 7 days. The release behavior exhibited two patterns: an initial burst of 40% for 7 h and the sustained release of 64% for 2 days.

### 2.3. In Vitro Cell Proliferation Ratio

**Figure 4:**
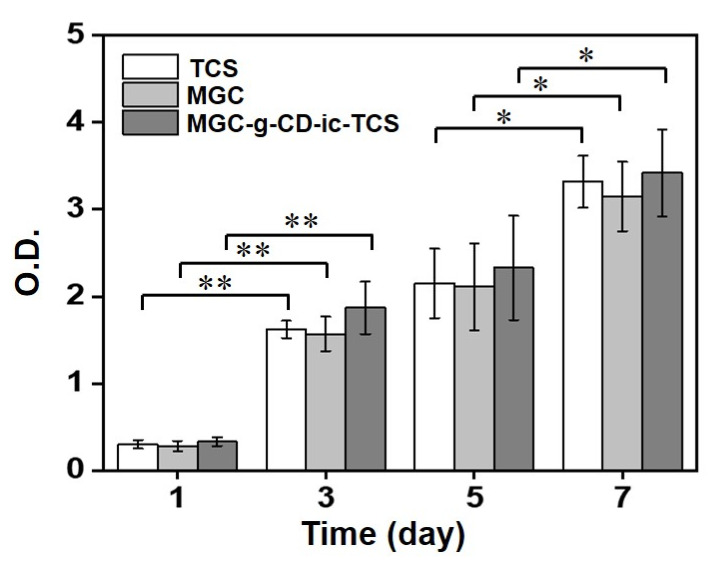
In vitro cell proliferation of L-929 cells cultured on TCS, MGC, and MGC-g-CD-ic-TCS for 1, 3, 5, and 7 days. This test was performed in triplicate (*n* = 3; * *p* < 0.05, ** *p* < 0.01).

The cell proliferation test of TCS, MGC and MGC-g-CD-ic-TCS was performed at 37 °C for 7 days using a cell counting kit-8 (CCK-8) assay (Figure 4). A gradual increase in cell proliferation was observed in all samples for 7 days. TCS is known to have cytocompatibility below the concentration of 5 µg/mL, but above the concentration, it induces cytotoxicity by stimulating apoptosis [27,28]. Considering this fact, the concentration (1.8 µg/mL) of TCS used in this study is within biocompatible range. Although there was no marked difference of the cell proliferation ratio among the samples, MGC-g-CD-ic-TCS exhibited a higher cell proliferation ratio than TCS and MGC. In addition, the cell proliferation ratios of TCS, MGC and MGC-g-CD-ic-TCS at day 7 were 11.1-, 11.3-, and 10.4-fold higher than those at day 0. Some previous studies reported that photocured MGC hydrogels initiated by riboflavin can be used as biocompatible platforms for tissue engineering applications [11,12,13,14,29,30]. TCS has been reported to have toxicity against fish, algae, and aquatic organisms [24]. Hence, to reduce the side effects of TCS, hydrogels were introduced because they do not have direct contact with cells. Our results showed that MGC-g-CD-ic-TCS has a higher cell proliferation rate than TCS and MGC, indicating that the hydrogel is a safe material for controlling the cytotoxicity of TCS.

### 2.4. Antibacterial Activity

**Figure 5:**
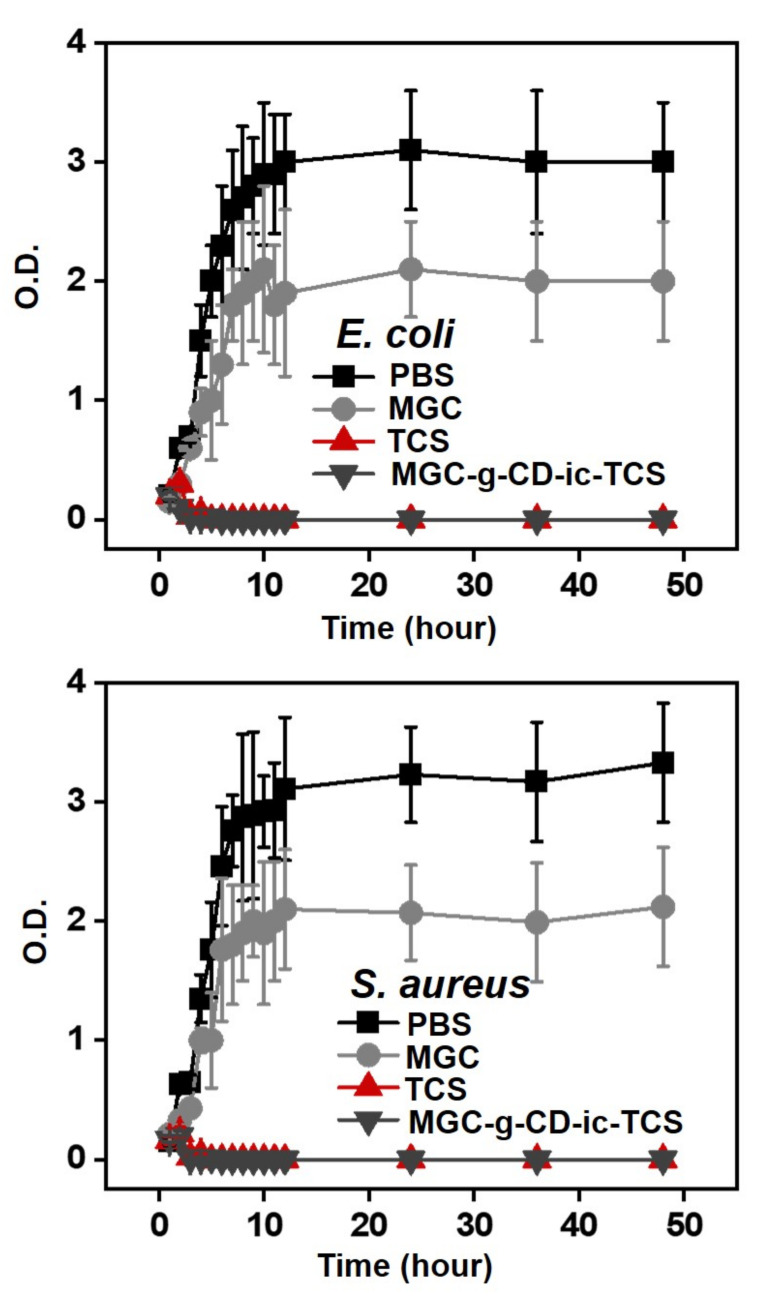
Antibacterial activity of MGC-g-CD-ic-TCS on *E. coli* and *S. aureus*, compared to PBS, MGC, and TCS. This test was performed in triplicate (*n* = 3).

The antibacterial activity of MGC-g-CD-ic-TCS was evaluated using *Escherichia coli* and *Staphylococcus aureus* (Figure 5). The *E. coli* and *S. aureus* are widely used as Gram-negative food-borne pathogens and Gram-positive pathogen, respectively [31]. The antibacterial activity of materials is deeply related to their surface charges and surface hydrophilic/hydrophobic natures [32]. We confirmed that MGC and MGC-g-CD-ic-TCS improve antibacterial activity against *E. coli* and *S. aureus*, as compared to PBS (Figure 5). It is well known that chitosan and its derivatives have good interaction with bacteria due to ionic interaction between the cationic charge of the polymers and the negative charge of bacteria [32,33]. The amine group of glycol chitosan (GC) is protonated when the bacterial medium is lower than pKa of 6.0 [34]. In the case of our study, the bacterial medium was almost 7.4, which indicated that MGC was not protonated. Therefore, the result of this study may be affected by the surface hydrophilic/hydrophobic nature of MGC. It has been reported that bacteria are well adhered to hydrophobic surface [33]. Based on this fact, the antibacterial activity of MGC is mainly dependent on its surface hydrophilicity. In addition to MGC, TCS improved antibacterial activity because it has a broad antimicrobial spectrum including bacteriostatic property as low concentration and induce cell death by disrupting bacteria membrane at high concentration [7,35]. The hydrogel with almost 40% of TCS release for 5 hours was found to have improved antibacterial activity [24]. From our result, together with the antibacterial activity of MGC, almost 30% of TCS release showed improved antibacterial activity. In addition, to accelerate antibacterial activity, TCS must have a concentration of above 5 µg/mL [28]. From this result, 1.8 µg of TCS containing MGC-g-CD exhibited excellent antibacterial activity. This reason can well explain that MGC-g-CD-ic-TCS is more effective against antibacterial activity than free TCS, due to the synergistic effect of MGC and TCS.

### 2.5. Adhesion Strength of Hydrogels to Tissues

**Figure 6:**
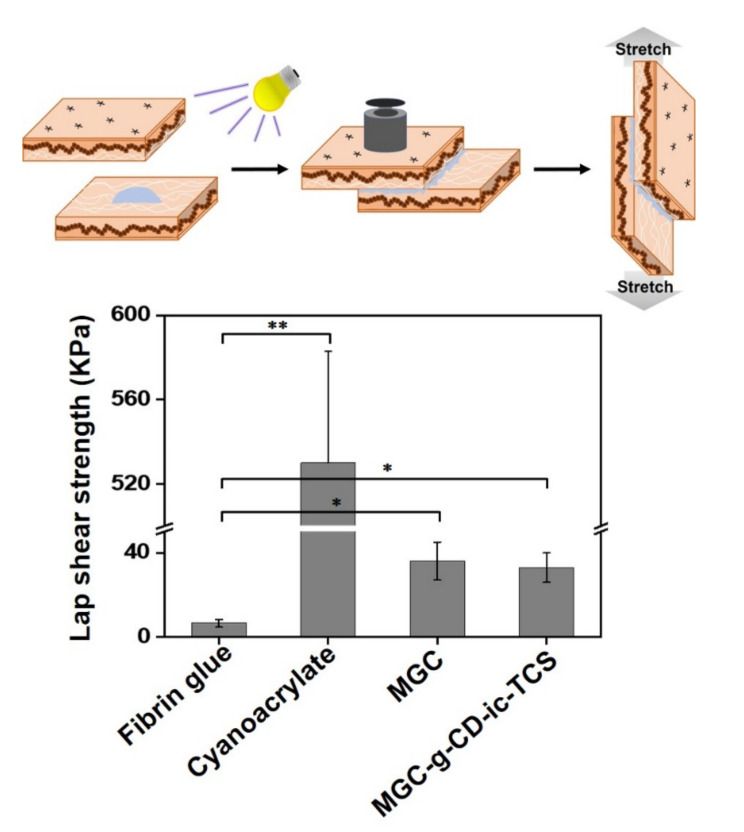
Lap shear strength of fibrin glue, cyanoacrylate, MGC, and MGC-g-CD-TCS. This test was performed in triplicate (*n* = 3; * *p* < 0.05, ** *p* < 0.01).

The adhesion strength of MGC-g-CD-ic-TCS was measured using a standard test method (ASTM F2255-05) for evaluating the tissue adhesive strength and the result was compared to those of fibrin glue, cyanoacrylate, and MGC, as shown in Figure 6. In hydrogel systems with non-surface-active groups, interactions including van der Waals forces and hydrogen bonding to soft tissues occur, which leads to weak binding ability. Meanwhile, positive-charged chitosan can contribute to stronger binding to tissue through multiple ionic interactions than neutral-charged polymers [36,37]. The results demonstrated that fibrin glue, cyanoacrylate, MGC, and MGC-g-CD-ic-TCS have 6.5 kPa, 530 kPa, 36 kPa, and 33 kPa of adhesive ability, respectively. The cyanoacrylate has a superior adhesiveness, but it is not suitable for tissue adhesion due to its toxicity, whereas fibrin glue is used as a control bio-adhesive due to its good biocompatibility. MGC, and MGC-g-CD-ic-TCS, had a stronger adhesion to tissue than fibrin glue due to its amine group can induce ionic interaction with tissues, resulting in adhesive strength more than five times.

### 2.6. Observation of Gross Appearances of Skin Incisions

**Figure 7:**
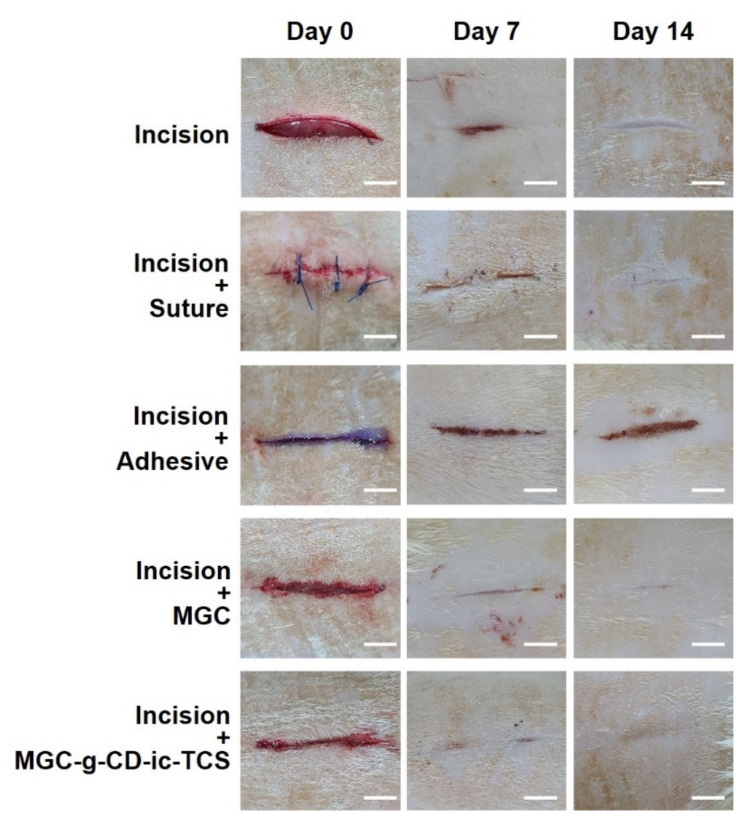
Gross appearances of untreated incision, and incisions treated with suture (incision + suture), cyanoacrylate (incision + adhesive), MGC (incision + MGC), and MGC-g-CD-ic-TCS (incision + MGC-g-CD-ic-TCS). The incisions were observed at day 0, 7, and 14. The white scale bars indicate 1 cm.

For improving the bioavailability of TCS in wound healing, its water solubility and release behavior must be advanced. β-CD-grafted MGC-based hydrogel can meet the characteristics by inclusion of complex formation between the ring molecule and the drug as shown in Figure 2 and Figure 3. In addition, MGC-g-CD-ic-TCS was found to be cytocompatibility and has a superior tissue adhesive compared to fibrin glue, as shown in Figure 4, Figure 5 and Figure 6. Figure 7 shows the gross appearances of skin incisions treated with suture, cyanoacrylate adhesive, MGC, and MGC-g-CD-ic-TCS for 14 days, as compared with that of sham. On day 7, the incisions in all experimental groups appeared to heal. Among the groups, more rapid healing was observed in the incisions treated with MGC and MGC-g-CD-ic-TCS. On day 14, scab was still observed in the sham, and suture-, and cyanoacrylate-treated incisions. On the other hand, MGC and MGC-g-CD-ic-TCS seemed to accelerate wound healing. On day 14, the incisions in the other groups remained scar except MGC-g-CD-ic-TCS-treated incision. Among incision, incision + suture, incision + adhesive, and incision + MGC, the MGC-treated sample showed the smallest scar. The incision + adhesive sample still exhibited scab due to the toxicity of cyanoacrylate.

### 2.7. Histological Evaluations of Skin Incisions

**Figure 8:**
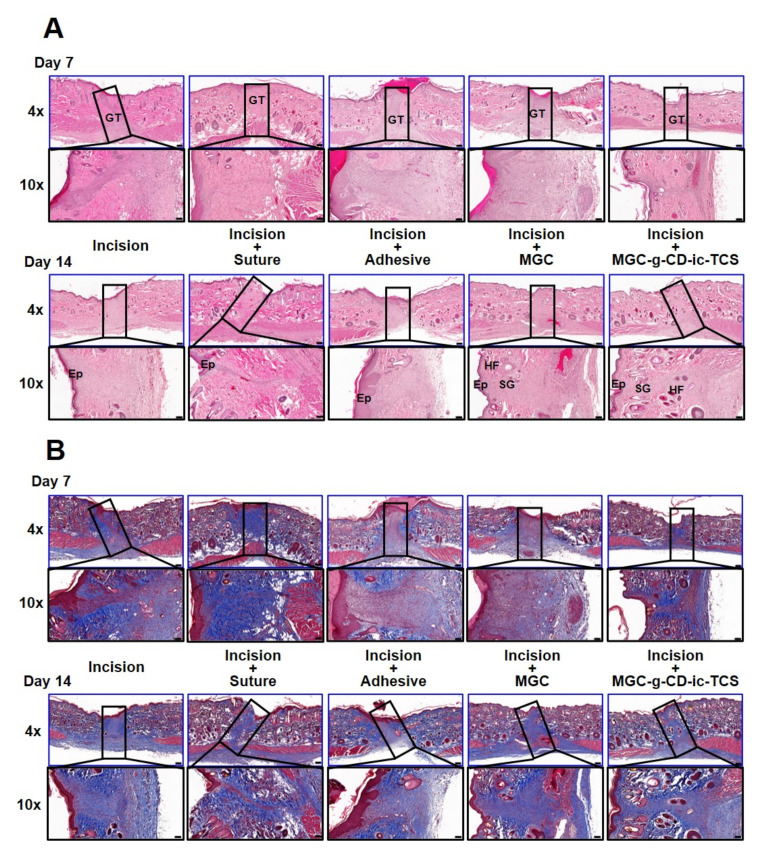
(**A**) H&E-stained and (**B**) MT-stained images of untreated incision, and incisions treated with suture (incision + suture), cyanoacrylate (incision + adhesive), MGC (incision + MGC) and MGC-g-CD-ic-TCS (incision + MGC-g-CD-ic-TCS) after 7 and 14 days. The slides were observed at 4× (200 µm) and 10× (100 µm), respectively. At 4× images, the rectangle boxes indicate incised part. GT-granulation tissue; EP-epithelial layer; HF-hair follicles; SC-sebaceous glands.

To further investigate the histological changes in the incisions of all groups, hematoxylin and eosin (H&E) and Masson’s trichrome (MT) staining were performed at days 7 and 14, respectively (Figure 8). From the H&E results (Figure 8A), at day 7, granulation tissue was newly formed in the incisions of all groups together with the infiltration of inflammatory cells. In addition, hair follicles and sebaceous glands began to develop in the MGC-g-CD-ic-TCS-treated incision. At 14 days, accelerated wound healing was observed in the incision + suture sample. However, incision, incision + suture, and incision + adhesive samples exhibited a thick epidermal layer, indicating abnormal wound healing. In particular, the proliferative epithelium was still observed in the incision + adhesive sample. In the incision + MGC and incision + MGC-g-CD-ic-TCS samples, an epidermis of normal thickness was formed. Hair follicles and sebaceous glands were first observed in the incision + MGC samples. In addition, hair follicles and sebaceous glands were mostly found in the MGC-g-CD-ic-TCS-treated incision.

Collagen deposition in the incisions was evaluated by MT staining (Figure 8B). Compared with incision + adhesive and incision + MGC samples, the incision, incision + suture, and incision + MGC-g-CD-ic-TCS showed active collagen production. However, on day 14, the incision, incision + suture, incision + adhesive, and MGC samples still seemed to stay in the proliferative stage. However, normal wound healing was observed in the incision + MGC-g-CD-ic-TCS sample. These results can be ascribed by the cocktail effect of MGC and TCS, because MGC and TCS can provide appropriate tissue junction and antibacterial/antifungal effect, respectively [7,35,38]. An animal study demonstrated that MGC-g-CD-ic-TCS accelerates re-epithelialization and wound remodeling in incision, compared with suture, cyanoacrylate, and MGC. Moreover, MGC induced a greater wound healing process than suture and cyanoacrylate. These results attribute to the adjustment of M1 and M2 polarization.

**Figure 9:**
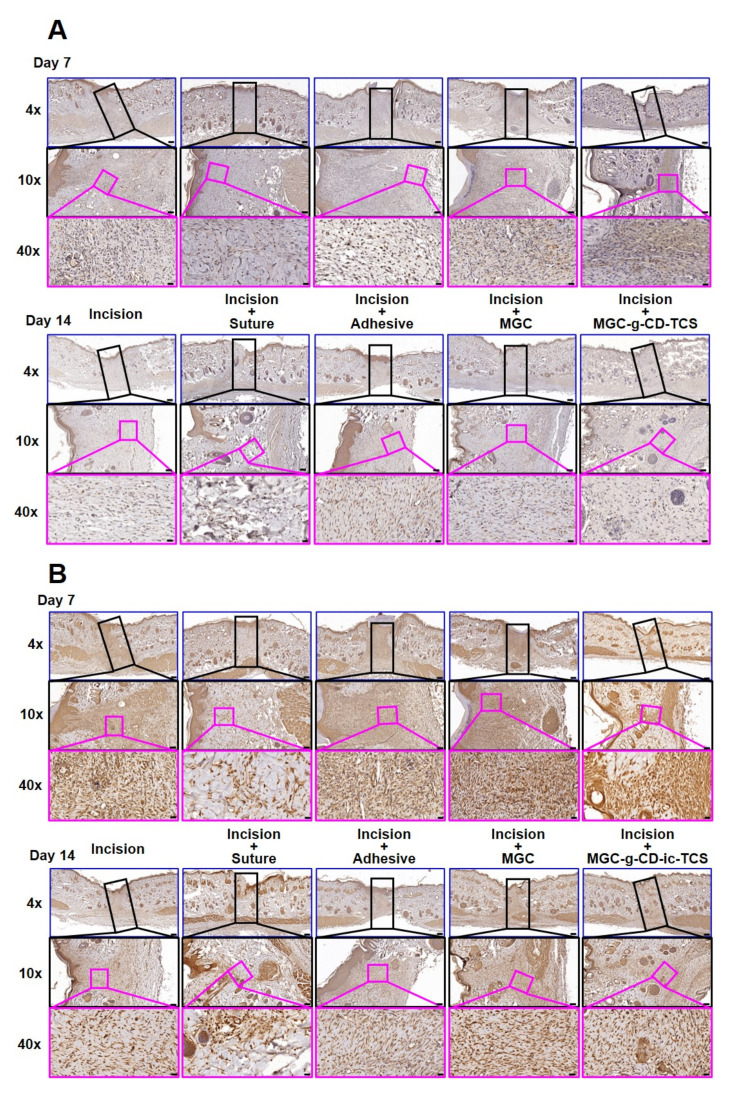
(**A**) Performed M1-specific anti-iNOS antibody and (**B**) M2-specific anti-mannose receptor antibody immunostained images of untreated incision, and incisions treated with suture (incision + suture), cyanoacrylate (incision + adhesive), MGC (incision + MGC), and MGC-g-CD-ic-TCS (incision + MGC-g-CD-ic-TCS) after 7 and 14 days. The slides were observed at 4× (200 µm), 10× (100 µm) and 40× (20 µm), respectively. At 4× images, the black rectangle boxes indicate incised part. At 10× images, the pink rectangle boxes indicate M1-specific anti-iNOS antibody and M2-specific anti-mannose receptor antibody immunostained images.

**Figure 10:**
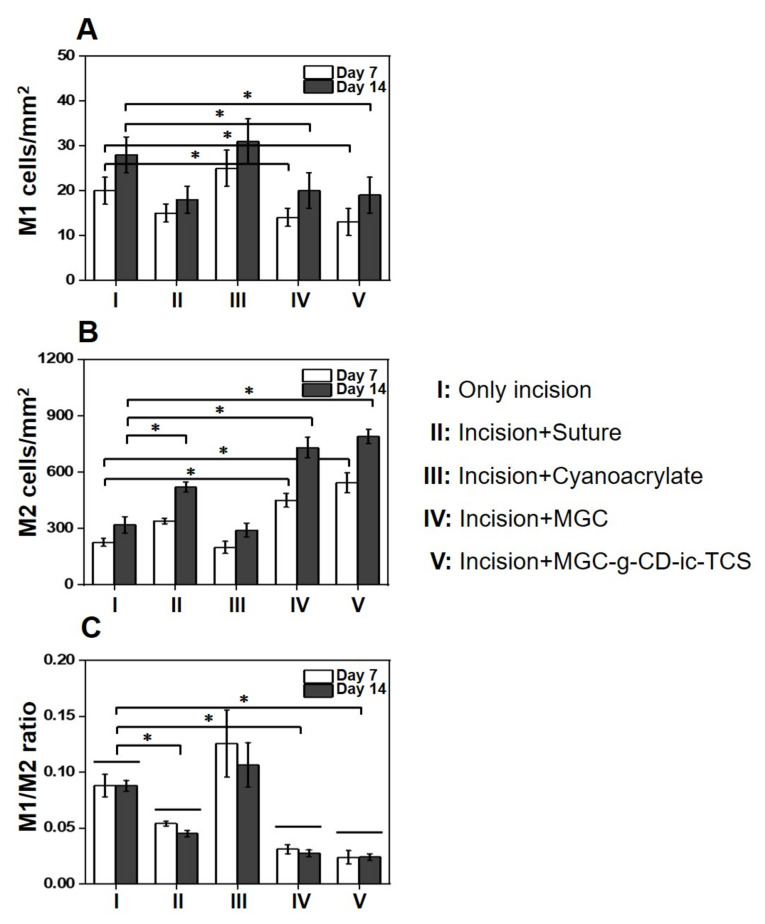
(**A**) M1, (**B**) M2, and (**C**) M1/M2 ratio of untreated incision, and incisions treated with suture (incision + suture), cyanoacrylate (incision + adhesive), MGC (incision + MGC), and MGC-g-CD-ic-TCS (incision + MGC-g-CD-ic-TCS) after 7 and 14 days (*n* = 3, * *p* < 0.05).

To investigate the infiltration of macrophages on days 7 and 14, we performed M1-specific anti-iNOS antibody and M2-specific anti-mannose receptor antibody immunostaining, as shown in Figure 9A. Over the time, MGC and MGC-g-CD-ic-TCS decreased the number of iNOS-positive cells and increased mannose receptor-positive cells. On day 7, the numbers of iNOS-positive cells in the incision, incision + suture, incision + adhesive, incision + MGC, and incision + MGC-g-CD-ic-TCS samples were 20, 16, 25, 15, and 14 cells/mm^2^, respectively, and the numbers of mannose receptor-positive cells were 280, 550, 260, 500, and 600 cells/mm^2^, respectively. At day 14, the M1/M2-related cells increased in the five groups, which exhibited 28, 18, 32, 20, and 19 cells/mm^2^ in the case of iNOS-positive cells, and 310, 650, 290, 750, and 820 cells/mm^2^ in the case of mannose receptor-positive cells. To further investigate the effect of M1 and M2 macrophages on wound healing, M1, M2, and M1/M2 ratio were calculated (Figure 9B). As shown in Figure 10A, incision + suture, incision + MGC, and incision + MGC-g-CD-ic-TCS samples exhibited a significantly fewer M1 cell numbers than incision and incision + cyanoacrylate. On the contrary to this, incision and incision + cyanoacrylate samples exhibited a significantly larger M2 cell number than the other samples (Figure 10B). Among all samples, the fewest M1 and the largest M2 cells were observed in incision + MGC-g-CD-ic-TCS. In addition, M1/M2 ratio tended to be similar in comparison with the M1 result (Figure 10C). However, on days 7 and 14, the increase of M1 and M2 cells in all samples may demonstrate the co-existence of inflammation, proliferation, and remodeling stages. These results imply that Incision + MGC-g-CD-ic-TCS provides a better wound healing process from inflammation to proliferative/remodeling stages.

The immunostaining results of M1 and M2 suggest that MGC-g-CD-ic-TCS more reasonably modulates the polarization of macrophages than suture, cyanoacrylate, and MGC. As noted in a previous study, chitosan modulates macrophage polarization toward M2 phenotype at late differentiation stages in in vitro cell experiment [38]. A quick phenotypic switching from M1 to M2 in wound healing process leads to a fast resolution of inflammatory response and an improved local tissue regeneration [39]. In addition, chitosan hydrogel applied in the wounds of mice, rats, and pigs accelerates collagen synthesis, re-epithelialization, and angiogenesis [40,41,42]. In addition, TCS is known to possess a broad spectrum of antibacterial properties because it can inhibit the synthesis of fatty acid required for the membrane and wall of bacteria at low concentration and disrupt the membrane of bacteria at high concentration [7,35,38]. The stage of wound healing is a complex process involving numerous cells and cytokines in the temporospatial relationship [43]. Therefore, it is natural that it is not coordinated into a single molecule. The purpose of this study is not tissue-engineered skin regeneration or soft tissue regeneration, but hydrogel-based skin adhesive, designed for closing skin wounds. TCS has already been impregnated on absorbable sutures due to its antibacterial effect and is widely used [44]. Therefore, the purpose of this study was to perform a basic release test to evaluate whether triclosan contained in hydrogel-based polymeric adhesive exhibits antibacterial or even antibacterial induced anti-inflammatory effect. Our histologic evaluation is also focused on managing the inflammatory process rather than the entire soft tissue regeneration. From the results obtained by us, it is believed that hydrogel-based chitosan itself made the microenvironment of epidermis and partial dermal tissue favorable for wound healing, and that triclosan suppressed bacterial infection and maintained optimal inflammation throughout the wound. In this study, we used CD-ic-TCS-grafted glycol chitosan as a main material for bio-adhesive with normal wound healing in incision. Glycol chitosan and TCS have the same characteristics of chitosan as a type of chitosan derivative and help to steadily progress wound healing; therefore, the positive wound healing effect of MGC-g-CD-ic-TCS is affected by glycol chitosan and TCS.

## 3. Materials and Methods

### 3.1. Materials

GC (≥60%, crystalline, Mw ≅ 585,000 g/mol) and glycidyl methacrylate (GM) were purchased from Sigma-Aldrich (St. Louis, MO, USA) for MGC preparation. β-CD-COOH (St. Louis, MO, USA) was used for MGC-g-CD preparation. Riboflavin 5′-monophosphate sodium salt (riboflavin; Santa Cruz, CA, USA) was used as a photoinitiator. 4-(4,6-Dimethoxy-1,3,5-triazin-2-yl)-4-methyl-morphoninium chloride (DMT-MM) as a condensing agent was supplied by Wako Pure Chemical Industries (Osaka, Japan). TCS obtained from Sigma-Aldrich (St. Louis, MO, USA) was used as an antibacterial agent. As a cyanoacrylate adhesive, Liquiband^®^ Surgical S (Advanced Medical Solutions Group Plc, Cheshire, UK) was used. MGC-g-CD was purified using a dialysis membrane tube (cut-off: 25 kDa; Spectrum Laboratories Inc., Rancho Dominguez, CA, USA). Mouse fibroblast L-929 cell line was supplied by the Korean Cell Line Bank (Seoul, Korea). Chemicals were used as received.

### 3.2. Preparation of MGC-g-CD-ic-TCS

MGC was prepared according to procedures from our previous studies [11,12,13,14]. Next, β-CD-COOH was conjugated to the amine group of MGC through amide bond using DMT-MM as a condensation agent. To a solution of MGC (1.70 µmol, 1 g) in water, aqueous β-CD-COOH solution (1.15 mmol, 2.11 g, 500 mL) was slowly dropped and reacted at room temperature for 3 days. After dialysis for 7 days, the cleaned solution was lyophilized at −90 °C until dry. TCS (1.15 mmol, 333 mg) was dissolved in acetone (2 mL) and gently dropped in aqueous MGC-g-CD solution (1.5 g, 500 mL) filled in a beaker (1 L) with a continuous stirring (350 rpm). To evaporate acetone, the cap of the vial was removed and kimwipes^®^ was covered to the entrance. The mixture was continuously stirred until acetone was perfectly evaporated and precipitate was eliminated by centrifugation at 1200 rpm. After lyophilization of the supernatant, MGC-g-CD-ic-TCS was characterized by analyses of ^1^H NMR, DSC, and storage/loss moduli. The ^1^H NMR analysis was performed using D_2_O. The DSC (TA Instruments; DSC Q2000; New Castle, DE, USA) was employed for analyzing the thermal behavior of MGC-g-CD-ic-TCS, which was monitored from 50 °C to 170 °C at 10 °C/min by heating the hydrogel (50 mg) onto an aluminum pan. The storage/loss moduli of MGC-g-CD-ic-TCS were measured using an AR 2000ex rheometer (TA instruments, New Castle, DE, USA) at 37 °C from 0 Hz to 10 Hz.

### 3.3. Preparation of MGC-g-CD-ic-TCS Hydrogel

The hydrogel was prepared on a clean bench. Aqueous MGC-g-CD-ic-TCS solution was filtered using a 0.22 µm syringe filter for the removal of microorganisms, and then lyophilized. To a solution of MGC-g-CD-ic-TCS (200 mg) in DPBS (3 mL), aqueous riboflavin (12 µM; DPBS) was added, and the sample was vortexed gently. MGC-g-CD-ic-TCS was stabilized in a refrigerator (4 °C) for stabilization before use. For hydrogelation, the hydrogel precursor solution was irradiated for 200 s using a blue light (430–485 nm, 2100 mW/cm^2^, light-emitting diode curing light, Foshan Keyuan Medical Equipment Co., Ltd., Foshan, China). The behavior was calculated by the ratio of swollen weight to initial weight at each time interval.

### 3.4. In Vitro Release Test

The release test was carried out according to a previously reported method [45]. A specific volume of MGC-g-CD-ic-TCS hydrogel (1 mL) was added in a dialysis bag (cut-off: 50,000 g/mol), and the drug-loaded sample was then immersed in PBS (10 mL, pH 7.4) containing 1% Tween-80 at 37 °C. At predetermined time intervals, 500 µL of PBS was extracted, and the same volume of fresh PBS was added. All extracted PBS solutions were examined by a UV-Vis spectroscopy (Multiskan^®^ Spectrum; Thermo Fisher Scientific; Waltham, MA, USA) at a wavelength of 282 nm.

### 3.5. In Vitro Cell Proliferation Assay

L-929 cell line was cultured with Eagle’s Minimal Essential Medium supplemented with FBS (5%), penicillin (100 units/mL) and streptomycin (100 µg/mL). For this experiment, cells cultured for 10 passages were used. The cell proliferation assay was performed on two hydrogel samples: TCS (TCS: 1.8 µg), MGC, and MGC-g-CD-ic-TCS (TCS: 1.8 µg). The hydrogel precursor solutions were immersed in a 96-well plate and photocured using a visible light irradiation for 200 s. After seeding the cells (1 × 10^4^ cells/well) on the hydrogels, they were incubated for 1, 3, 5, and 7 days in an incubator set at 37 °C and 5% CO_2_. In the case of TCS, the cells (1 × 10^4^ cells/well) were attached on the wells of 96-well plate for 3 h and TCS-containing media was treated. At each time interval, CCK-8 (100 µL) was added in wells filled with the hydrogels and incubated for additional 2 hours. The optical density of supernatants extracted from CCK-8-treated wells was measured at 450 nm using a microplate reader (SpectraMax^®^ i3; Molecular Devices, Sunnyvale, CA, USA).

### 3.6. Tissue Adhesive Strength Measurement

This test was performed using procedures based on ASTM F2255-05. The adhesive strength of MGC-g-CD-ic-TCS hydrogel was measured using a universal testing machine (Instron Model 3343, Norwood, MA, USA). Prior to the test, the fat layer of the skin, which was used for this study, was removed. MGC-g-CD-ic-TCS hydrogel precursor solution was applied on the surface of the cleaned skin with 10 × 10 mm^2^ and another skin was covered to the sample-coated skin during visible light irradiation for 200 s. The adhesive strength of the bonded skins was measured at a crosshead speed of 10 mm/min with a 100 *n* load cell. This test was performed on 10 samples. As a control adhesive, the adhesive strength of fibrin glue was measured under the same conditions.

### 3.7. Evaluation of Antibacterial Activity

*Escherichia coli* ATCC25922 (*E. coli*) and *Staphylococcus aureus* ATCC6538 (*S. aureus*) were used for evaluating the antibacterial activity of MGC and MGC-g-CD-ic-TCS (TCS: 1.8 µg) hydrogels. It was estimated by monitoring the optical density of the two bacteria at 600 nm. *E. coli* or *S. aureus* (10 µL mid-long phase; O.D. = 0.3 units) were inoculated in fresh nutrient LB broth medium containing the two hydrogels (60 mg/L per each hydrogel) in flasks. LB medium alone was used as a negative control. Afterward, the flasks were incubated at 37 °C with a continuous stirring at 150 rpm. The optical density was measured for 48 h at 1-h intervals.

### 3.8. In Vivo Animal Study

This study was approved by the Institutional Animal Care and Use Committee of Chonbuk National University (23 March 2020; No: CBNU 2020-031). Male Sprague Dawley rats (three-month-old, 300–350 g) was used and randomized into five groups on each time interval (7 and 14 days; *n* = 5). A constant length of incision was produced on the back of each rat and the incision depth was made including the dermis. A specific volume (150 µL) of cyanoacrylate, MGC, and MGC-g-CD-ic-TCS (TCS: 1.8 µg). were used for three groups, respectively. Other two groups include incision + no treatment and incision + suture. After treating MGC and MGC-g-CD-ic-TCS hydrogel precursor solutions into the incisions, visible light (blue light; 430–485 nm, 2100 mW/cm^2^, light-emitting diode curing light, Foshan Keyuan Medical Equipment Co., Ltd., Foshan, China) was irradiated for 120 s. At the predetermined time intervals, the mice of each group were anesthetized, the skins including the incisions were removed, and the mice were sacrificed. All skin tissue samples were used for histological evaluations.

### 3.9. Histological Evaluations

Removed skin tissues including incisions were immediately placed in 10% formalin solution. The fixed tissues were dehydrated using a series of ethanol solutions, and the dehydrated tissues were then embedded in paraffin for block preparation. A constant thickness of slide was sectioned (3 µm). As a control, the same thickness of paraffin slide using normal skin tissue was made. The tissue sections were treated with H&E and MT staining (Abcam: ab150686, Cambridge, UK). For immunostaining, deparaffinized tissue sections were treated using a microwave antigen-retrieval procedure with 0.01 M sodium citrate buffer. After blocking, the sections incubated with antibodies against iNOS (N-20, Santa Cruz Biochemical, Dallas, TX, USA) and mannose receptor (Abcam). For visualization, an HRP/DAB detection IHC kit (Abcam) was used according to the manufacturer’s instructions. These sections were observed using a slide scanner (Pannoramic MIDI; 3DHISTECH Ltd., Budapest, Hungary) and a panoramic viewer (Version 1.15.3; Pannoramic MIDI; 3DHISTECH Ltd., Budapest, Hungary) program.

### 3.10. Statistical Analysis

All quantitative data were expressed as the mean ± standard deviation. Statistical analysis was performed with one-way analysis of variance (ANOVA) using SPSS software (SPSS Inc., Chicago, IL, USA). A value of * *p* < 0.05 was considered statistically significant.

## 4. Conclusions

Here, we designed and prepared a MGC-g-CD-ic-TCS hydrogel system as an antibacterial tissue bio-adhesive. Inclusion complexes between MGC-g-CD and TCS were confirmed by ^1^H NMR and DSC analyses. Due to the migration of water molecules into its 3-D structure, the hydrogel was swollen, and this influenced the release behavior of TCS. MGC-g-CD-ic-TCS exhibited better tissue bonding strength than commercially used fibrin glue. In addition, the hydrogel showed high antibacterial activity against *E. coli* and *S. aureus* because of the cocktail effect of MGC and TCS. More importantly, MGC-g-CD-ic-TCS was associated with accelerated wound healing in vivo, suggesting its great potential as a tissue bio-adhesive.

## Data Availability

Data is contained within the article.

## Abbreviations

TCS	Triclosan
β-CD-COOH	Succinyl beta-cyclodextrin
MGC	Methacrylated glycol chitosan
^1^H NMR	Proton nuclear magnetic resonance
DSC	Differential scanning calorimetry
MGC-g-CD-ic-TCS	TCS-complexed β-CD-conjugated MGC
3-D	Three-dimensional
CCK-8	Cell counting kit-8
GC	Glycol chitosan
H&E	Hematoxylin and eosin
MT	Masson’s trichrome
GM	Glycidyl methacrylate
Riboflavin	Riboflavin 5′-monophosphate sodium salt
DMT-MM	4-(4,6-Dimethoxy-1,3,5-triazin-2-yl)-4-methyl-morphoninium chloride
